# Incidence, organ dysfunction and mortality in severe sepsis: a Spanish multicentre study

**DOI:** 10.1186/cc7157

**Published:** 2008-12-17

**Authors:** Jesús Blanco, Arturo Muriel-Bombín, Víctor Sagredo, Francisco Taboada, Francisco Gandía, Luís Tamayo, Javier Collado, Ángel García-Labattut, Demetrio Carriedo, Manuel Valledor, Martín De Frutos, María-Jesús López, Ana Caballero, José Guerra, Braulio Álvarez, Agustín Mayo, Jesús Villar, the Grupo de Estudios y Análisis en Cuidados Intensivos (G.R.E.C.I.A.)

**Affiliations:** 1 Critical Care Department, Nuevo Hospital Universitario Río Hortega, Calle Dulzaina s/n, 47012 Valladolid, Spain; 2 CIBER de Enfermedades Respiratorias (Instituto de Salud Carlos III), Carretera Soller Km. 12, 07110 Mallorca, Spain; 3 Critical Care Department, Hospital Clínico Universitario de Salamanca, Paseo de San Vicente 182, 37007 Salamanca, Spain; 4 Critical Care Department, Hospital Central de Asturias, Calle Celestino Villamil s/n, Oviedo, 33006 Asturias, Spain; 5 Critical Care Department, Hospital Clínico Universitario de Valladolid, Avenida Ramón y Cajal 3, 47005 Valladolid, Spain; 6 Critical Care Department, Hospital Río Carrión, Calle Donantes de Sangre s/n, 34005 Palencia, Spain; 7 Critical Care Department, Hospital General de Soria, Carretera de Logroño s/n, 42004 Soria, Spain; 8 Critical Care Department, Complejo Hospitalario de León, Calle Altos de Nava s/n, 24008 León, Spain; 9 Critical Care Department, Hospital de San Agustín, Camino Heros 4, Avilés, 33410 Asturias, Spain; 10 Critical Care Department, Hospital General Yagüe, Avenida del Cid Campeador 96, 09005 Burgos, Spain; 11 Critical Care Department, Hospital General de Segovia, Carretera de Avila s/n, 40002 Segovia, Spain; 12 Critical Care Department, Hospital Virgen de la Concha, Avenida Requejo 35, 49022 Zamora, Spain; 13 Critical Care Department, Hospital de Cabueñes, Calle de los Prados 395, Gijón, 33394 Asturias, Spain; 14 Critical Care Department, Hospital del Bierzo, Calle Médicos sin Fronteras 7, Ponferrada, 24411 León, Spain; 15 Statistics Department, School of Medicine (University of Valladolid), Avenida Ramón y Cajal 7, 47005 Valladolid, Spain; 16 Multidisciplinary Organ Dysfunction Evaluation Research Network, Research Unit, Hospital Universitario Dr. Negrin, Barranco de la Ballena s/n, 35010 Las Palmas de Gran Canaria, Spain; 17 Keenan Research Center, St. Michael's Hospital, 30 Bond Street, Toronto, Ontario M5B 1W8, Canada

## Abstract

**Introduction:**

Sepsis is a leading cause of admission to non-cardiological intensive care units (ICUs) and the second leading cause of death among ICU patients. We present the first extensive dataset on the epidemiology of severe sepsis treated in ICUs in Spain.

**Methods:**

We conducted a prospective, observational, multicentre cohort study, carried out over two 3-month periods in 2002. Our aims were to determine the incidence of severe sepsis among adults in ICUs in a specific area in Spain, to determine the early (48 h) ICU and hospital mortality rates, as well as factors associated with the risk of death.

**Results:**

A total of 4,317 patients were admitted and 2,619 patients were eligible for the study; 311 (11.9%) of these presented at least 1 episode of severe sepsis, and 324 (12.4%) episodes of severe sepsis were recorded. The estimated accumulated incidence for the population was 25 cases of severe sepsis attended in ICUs per 100,000 inhabitants per year. The mean logistic organ dysfunction system (LODS) upon admission was 6.3; the mean sepsis-related organ failure assessment (SOFA) score on the first day was 9.6. Two or more organ failures were present at diagnosis in 78.1% of the patients. A microbiological diagnosis of the infection was reached in 209 episodes of sepsis (64.5%) and the most common clinical diagnosis was pneumonia (42.8%). A total of 169 patients (54.3%) died in hospital, 150 (48.2%) of these in the ICU. The mortality in the first 48 h was 14.8%. Factors associated with early death were haematological failure and liver failure at diagnosis, acquisition of the infection prior to ICU admission, and total LODS score on admission. Factors associated with death in the hospital were age, chronic alcohol abuse, increased McCabe score, higher LODS on admission, ΔSOFA 3-1 (defined as the difference in the total SOFA scores on day 3 and on day 1), and the difference of the area under the curve of the SOFA score throughout the first 15 days.

**Conclusions:**

We found a high incidence of severe sepsis attended in the ICU and high ICU and hospital mortality rates. The high prevalence of multiple organ failure at diagnosis and the high mortality in the first 48 h suggests delays in diagnosis, in initial resuscitation, and/or in initiating appropriate antibiotic treatment.

## Introduction

Sepsis is among the leading causes of admission to intensive care units (ICUs). Care for patients with sepsis represents a great economic burden [1] as extraordinary resources are devoted to developing and evaluating potential treatments as well as to studying the systemic inflammatory response and multiple organ failure that are characteristic of severe sepsis. The absence of clear definitions and diagnostic criteria for sepsis has hindered the advancement of epidemiological and clinical knowledge about this condition [2]; thus, clinical and therapeutic studies have often compiled data that are difficult to compare and extrapolate to clinical practice.

A review of studies evaluating the epidemiology of sepsis shows a very high prevalence, both among all hospitalised patients (one-third) and among those admitted to ICUs (over 50%). More than half of all septic patients develop severe sepsis and a quarter develop septic shock; thus, 10% to 15% of all patients admitted to ICUs develop septic shock [3]. The incidence of sepsis in studies reported in the last 10 years ranges from 9% to 37% of all patients admitted to the ICU [4-8]. The overall incidence of sepsis is approximately 300 cases/10^5 ^inhabitants/year in the USA [9]. The overall incidence of sepsis reported in Spain is 367 cases/10^5 ^inhabitants/year, including 104 cases of severe sepsis/10^5 ^inhabitants/year and 44 cases of sepsis attended in the ICU/10^5 ^inhabitants/year [10]. Martin *et al. *retrospectively documented 10,319,418 cases of sepsis among 750 million patients hospitalised in the USA between 1979 and 2000 [1]. A total of 27.1% of all patients admitted to ICUs in England, Wales, and Northern Ireland between 1995 and 2000 met the criteria for severe sepsis during the first 24 h after admission [11]. Another study found the incidence of septic shock among patients admitted to ICUs between 1993 and 2000 was 8.2% [12]. In recent years, the reported incidence of severe sepsis in patients admitted to ICUs ranged from 11.8% to 16.6% [13,14]. Of all episodes of infection recorded in ICUs, 28% are associated with sepsis, 24% with severe sepsis, and 30% with septic shock [15].

Published mortality rates for sepsis range from 28% to 56% [4-8]. The most recently published series report mortality rates ranging from 28% to 30% in mixed ICU populations [9,13,16]; 30-day mortality rates range from 32.4% to 35.5% [13,14], and in-hospital mortality may be as high as 47% [11]. Various factors have been associated with increased risk of death: inappropriate antibiotic use, the presence of comorbidities and shock, the need for vasoactive agents, multiple organ dysfunction, neutropoenia, *Candida *or *Enterococcus *bacteraemia, and intra-abdominal, pulmonary, or unknown location infection [9,11,17].

We present the first extensive dataset on the epidemiology of severe sepsis treated in the ICU in Spain. The study design and data collection were carried out prior to the publication of the Surviving Sepsis Campaign (SSC) Guidelines for management of severe sepsis and septic shock [18] and before the approval of activated protein C use in Spain.

## Materials and methods

### Primary objectives

Our primary goals were: (i) to determine the incidence of severe sepsis among adults in ICUs at general hospitals in a specific geographical health care area of Spain, and (ii) to determine the early (48 h), ICU, and hospital mortality rates as well as the factors associated with the risk of death in these patients.

### Secondary objectives

Our secondary outcomes were (i) to determine the frequency of different types of organ dysfunction when severe sepsis is diagnosed; (ii) to study the evolution of organ dysfunction throughout the septic process; (iii) to determine the types of infection (acquisition and microbiology) involved, and (iv) to determine the frequency of other factors associated with severe sepsis, including both clinical (comorbidities, shock) and therapeutic factors.

### Design and data collection

This was a prospective, observational, multicentre, cohort study. The study protocol was approved by the Ethics Committee of the coordinating centre (Hospital Universitario Río Hortega, Valladolid, Spain). This approval is legally valid in Spain for all others participating centres. The study was considered an audit and informed consent was waived. After the inclusion, all patients (or their legal representatives) were asked for informed written consent for blood withdrawal of a 10 ml sample for further analysis. The study was carried out over two 3-month periods, from 1 April to 30 June 2002, and from 1 October to 30 December 2002, in 14 ICUs in 13 Spanish hospitals (10 in the region of Castilla y León and 3 in the region of Asturias) belonging to the public healthcare network. All patients were screened for severe sepsis on ICU admission and daily thereafter. We recorded all consecutive episodes of severe sepsis, including both cases in which the episode was the reason for admission to the ICU and episodes diagnosed in patients already admitted to the ICU for any other reason.

All data were collected on standardised forms by the physicians (members of the Grupo de Estudios y Análisis en Cuidados Intensivos (GRECIA)) responsible for the study in each ICU (see Additional file 1 for a list of members of the GRECIA group); all were specialised in intensive care medicine and had extensive experience in the diagnostic criteria for severe sepsis. Data forms were sent to a custom-built Access database (Microsoft, Redmond, WA, USA) at the coordinating centre. All data related to physiological and biological variables were checked against standardised ranges by the medical staff at the coordinating centre. Inconsistent or extreme values were thoroughly checked and corrected before analysis. Variables recorded included the McCabe score [19] for the severity of underlying conditions and known comorbidities before severe sepsis developed. Clinical and laboratory data to enable the Acute Physiology and Chronic Health Evaluation (APACHE) II Score [20] to be calculated were collected the first 24 h after ICU admission. The Logistic Organ Dysfunction System (LODS) [21] score was calculated at day 0 (D0), on the basis of the data recollected from inclusion to 24:00 of the same day. The Sepsis-related Organ Failure Assessment (SOFA) [22] score was calculated on days 1, 3, 7, 11, and 15 (D1, D3, D5, D11, D15) from inclusion to evaluate the progression of multiple organ dysfunction. In all cases, unavailable clinical or laboratory data were assigned a value of 0 in the analysis. Neurological status was determined using the Glasgow Coma Scale (GCS) prior to sedation.

Patients were considered to have an infection if this was microbiologically documented according to the standard definitions of the Centers for Disease Control and Prevention (CDC) [23] or at least clinically suspected requiring evidence such as the presence of white blood cells in a normally sterile body fluid, perforated viscus, chest X-ray consistent with pneumonia and associated with purulent tracheal secretion, or a clinical syndrome associated with a high probability of infection. Infection was classified according to the mode of acquisition (community, hospital, or ICU), to the method of diagnosis (suspected, clinically documented through imaging or surgical findings, or microbiologically documented), to the microorganisms responsible when these were isolated, and to the organ(s) affected. We recorded whether the initial antibiotic therapy was appropriate according to the antibiogram for the microorganisms responsible when these were isolated. We recorded the dates of admission to the hospital and to the ICU, the date and time of inclusion in the study estimated from the time the attending physician considered that the patient fulfilled the criteria for severe sepsis, and the date of death if the patient died or the date of discharge from the ICU and from the hospital if the patient survived.

### Calculation of the accumulated incidence

Each hospital belonging to the Public Health Care Network in Spain provides medical care for a specific geographical healthcare area with a known population. To avoid bias, we calculated the overall incidence for the population from only those healthcare areas in which all existing ICUs participated in the study. Thus, data from 11 ICUs in 10 hospitals corresponding to 10 healthcare areas were used. The total number of residents ≥ 18 years of age in these healthcare areas was obtained from the 2001 census published by the Spanish National Statistics Institute (INE) [24]. Patients were included into the study if they were admitted to an ICU for severe sepsis during the study period or if they presented with an episode of severe sepsis during the study period after admission to the ICU for any reason. Patients ≤ 18 years of age were excluded. Patients admitted for ischaemic heart disease, cardiac arrhythmia and heart block were excluded since they were not considered at risk for sepsis. However patients undergoing open heart surgery were considered in our patient population. Definitions for ICU type, patient categories, comorbidities, systemic inflammatory response syndrome, sepsis, severe sepsis, septic shock and organ dysfunction are outlined in Additional file 2.

### Statistical analysis

Quantitative data are described as mean ± standard deviation (SD), medians and percentiles. Comparisons were performed using the Student t test or the Mann-Whitney U test, as appropriate; values of p < 0.05 were considered significant. Categorical data were analyzed by means of frequencies, percentages, and their confidence intervals. The estimated accumulated incidence of severe sepsis for the healthcare area was expressed as cases per 100,000 inhabitants per year. Patients were analyzed according to: (a) onset of severe sepsis (at admission or during hospitalisation); (b) acquisition site of severe sepsis (community, hospital, ICU); (c) type of ICU admission (medical, surgical, traumatological); (d) microbiological type and site of infection; (e) McCabe score; (f) LODS score on day 0 (D0); (g) SOFA score on day 1 (D1), day 3 (D3), day 7 (D7), day 11 (D11), and day 15 (D15). The progression of multiple organ dysfunction was assessed by sequentially calculating the SOFA score in survivors and non-survivors on days 1, 3, 7, 11, and 15 from the time of diagnosis. The standardised area under the curve (AUC) of the SOFA score over time was calculated for survivors and non-survivors and then compared using the Student ttest. Risk factors associated with ICU and hospital mortality were analyzed univariately and then multivariately by logistic regression; the degree of association with mortality was expressed as independent factors by means of odds ratios with their corresponding 95% confidence intervals. The statistical analysis was carried out by the staff of the coordinating centre and by a professor at the Department of Biostatistics of the Medical School at the University of Valladolid, who did not participate in collecting the data. All analyses were performed using SAS (version 8.02, SAS Institute, Cary, NC, USA) and SPSS (version 11.0.1, SPSS, Chicago, IL, USA) statistical software.

## Results

A total of 4 University hospitals, 3 University-associated hospitals and 6 Community hospitals participated in the study, with a total of 164 ICU beds and 14 ICUs (1 Medical, 12 Medical/Surgical and 1 Cardiac Surgical). Of these 14 ICUs, 9 had coronary units.

### Incidence of severe sepsis

**Figure 1:**
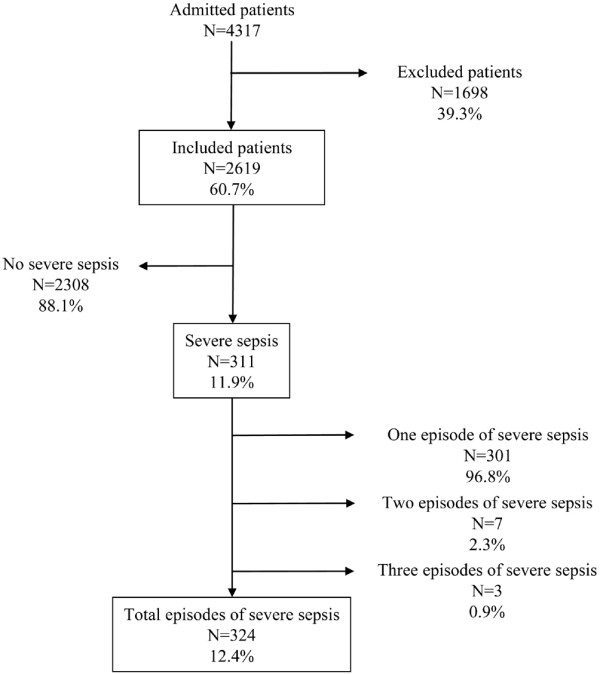
**Episodes of severe sepsis recorded in the patients admitted to the ICUs**.

During the study period, a total of 4,317 patients were admitted to the participating ICUs; 1,698 (39.3%) of these were excluded, including 1,658 (38.4%) because they had non-infectious heart problems and 40 (0.9%) were under 18 years of age. Thus, 2,619 patients (60.7%) were eligible for the study; 311 (11.9%; 95% CI 10.6 to 13.1) of these presented at least 1 episode of severe sepsis. A total of 324 (12.4%; 95% CI 11.1 to 13.6) episodes of severe sepsis were recorded: 80.8% of the episodes were diagnosed at or before ICU admission and the remaining 19.2% occurred in patients already in the ICU for various reasons. Seven patients presented two consecutive episodes of severe sepsis and three patients presented three consecutive episodes during their stay in the ICU (Figure 1).

A total of 246 episodes of severe sepsis were attended in the ICUs of the 10 hospitals that were considered for the estimation of the incidence in the general population. In 2001, a total of 1,946,130 inhabitants over 18 years of age resided in the geographical area assigned to these hospitals; 895,593 (46%) of these lived in urban areas, while the rest lived in rural areas [24]. The estimated accumulated incidence for the population was 25 cases of severe sepsis attended in ICUs per 100,000 inhabitants residing in the healthcare area per year.

The characteristics of the patients presenting at least one episode of severe sepsis that were included in the study are shown in Table 1. The mean (SD) APACHE II score was 25.5 (± 7.1) (median = 25). The mean (SD) LODS score was 6.3 (± 3.6) (median = 6); the mean (SD) SOFA score on the first day was 9.6 (± 3.7) (median = 10). Upon admission, roughly a quarter of the patients had one, two or three organ failures respectively and 78.1% had more than two organ failures.

**Table 1:** Demographic and clinical characteristics of the 311 patients at the time of diagnosis of the first episode of severe sepsis. See definitions in the text and list of abbreviations for meaning

		**Parameter**	**95% CI**
	Median	IQR	
Age (years)	68	54.9 to 74.5	

	No.	%	
Sex (male)	208	66.9	61.3 to 72.1
McCabe score:			
0	81	26	21.2 to 31.2
1	131	42.1	36.5 to 47.8
2	63	20.3	15.9 to 25.1
3	36	11.6	8.2 to 15.7
Category:			
Medical	179	57.6	51.9 to 63.1
Urgent surgery	93	29.9	24.9 to 35.3
Scheduled surgery	23	7.4	4.7 to 10.9
Traumatological	16	5.1	3.0 to 8.2
Origin:			
Medical and Surgical ward	167	53.7	48.0 to 59.0
Emergency department	67	21.5	12.5 to 21.0
Operating room	51	16.4	17.1 to 26.5
Other	26	8.3	5.5 to 12.0
Comorbidities:			
Chronic respiratory failure	46	14.8	11.0 to 19.2
Immunodeficiency	41	13.2	9.6 to 17.5
Risk of bleeding	31	10	6.9 to 13.8
Chronic alcoholism	25	8	5.3 to 11.6
Chronic renal failure	23	7.4	4.7 to 10.9
IDDM	22	7.1	4.5 to 10.5
Metastatic cancer	17	5.5	3.2 to 8.6
Chronic heart failure	15	4.8	2.7 to 7.8
Chronic liver failure	14	4.5	2.5 to 7.4
AIDS	4	1.3	0.4 to 3.3
No. of comorbidities:			
None	140	45	39.4 to 50.7
One	114	36.7	31.3 to 42.3
Two	46	14.8	11.0 to 19.2
Three or more	11	3.5	1.8 to 6.2
Organ failure:			
Respiratory	233	74.9	69.7 to 79.6
Shock	180	57.9	52.1 to 63.4
Cardiovascular	158	50.8	45.1 to 56.5
Renal	124	39.9	34.4 to 45.6
Haematological	69	22.2	17.7 to 27.2
Liver	40	12.9	9.3 to 17.1
Neurological	37	11.9	8.5 to 16.0
No. of organ failures:			
One	68	21.9	17.4 to 26.9
Two	86	27.7	22.8 to 32.0
Three	81	26	21.3 to 31.3
Four	39	12.5	9.1 to 16.7
Five or more	37	11.9	6.6 to 16.0

	Median	Mean (SD)	
APACHE II score (D1)	25	25.5 (± 7.1)	24.5 to 26.2
LODS score (D0)	6	6.3 (± 3.6)	5.9 to 6.7
SOFA score (D1)	10	9.6 (± 3.7)	9.2 to 10.0

APACHE II, Acute Physiology and Chronic Health Evaluation II; CI, confidence interval; D0: day 0; D1: day 1; IDDM, insulin-dependent diabetes mellitus; IQR: interquartile range; LODS, Logistic Organ Dysfunction System; SD, standard deviation; SOFA, Sepsis-related Organ Failure Assessment.

### Characteristics of the infection

The infections were acquired in the community in 51.5%, in the ICU in 19.2%, and in other areas of the hospital in 29.3%. Lungs were the predominant site of infection (44.8%), followed by the abdomen (31.5%), urinary tract (6.2%), central venous catheter (4.6%), soft tissues (3.1%) and surgical wounds (3.1%). The most common clinical diagnosis related to an episode of severe sepsis was pneumonia (139 episodes, 42.9%, 95% CI 37.4 to 48.5). Of these, 63 episodes were community-acquired pneumonia (19.4%) and 76 episodes were nosocomial pneumonia (23.5%). The second diagnosis was peritonitis not secondary to surgical intervention (47 episodes, 14.5%, 95% CI 10.9 to 18.8) followed by non-surgical infection of the digestive tract in 26 episodes (8%, 95% CI 5.3 to 11.5), bacteraemia associated with abdominal infection in 16 episodes (4.9%, 95% CI 2.8 to 7.9) and urinary tract infection in 14 episodes (4.3%, 95% CI 2.4 to 7.1); other diagnoses were less frequent.

A microbiological diagnosis of the infection was reached in 209 episodes of sepsis (64.5%). Table 2 shows the frequency of the different sites of infection and of the different microorganisms isolated. The diagnosis was reached clinically in a total of 82 episodes (25.3%), based on the clinical presentation and imaging findings in 14.5% and on surgical findings in 10.8%. In the remaining 33 episodes (10.2%), the diagnosis was highly suspicious.

**Table 2:** Frequencies of identified microorganisms and sites of isolation

	**%**
Gram negative bacilli (GNB) (n = 129)	50
*E. coli*	37.2
*Pseudomonas aeuruginosa*	20.9
*Acinetobacter baumanii*	10.9
*Legionella pneumophila*	7.8
*Klebsiella pneumoniae*	3.1
*Proteus mirabilis*	3.1
*Serratia marcescens*	2.3
*Haemophilus influenzae*	2.3
Gram positive cocci (GPC) (n = 104)	40.3
*Staphylococcus aureus*	32.7
*Streptococcus pneumoniae*	21.2
*Enterococcus faecalis*	9.6
*Staphylococcus epidermidis*	7.7
Coagulase negative Staphylococcus	6.7
Others	22.1
Fungi (n = 15)	5.8
*Candida albicans*	66.7
Candida spp	20
*Pneumocystis carinii*	13.3
Other (n = 10)	3.9
*Clostridium perfringens*	50
Corinebacterium	20
*Clostridium ramosum*	10
*Neisseria meningitidis*	10
Herpes Zoster virus	10
	
GNB sites:	
Tracheal aspirations	33.8
Blood cultures	23.3
Abdomen	13.7
Serology	7
Surgical wound	5.4
Urine	5.4
Skin	3.1
Others	3.3
GPC sites:	
Tracheal aspirations	33.7
Blood cultures	31.7
Abdomen	10.6
Surgical wound	7.7
Serology for pneumococci	6.8
Skin	2.9
Catheter	2.9
Others	4.7
Fungi sites:	
Tracheal aspiration	26.7
Urine	26.7
Bronchoalveolar lavage	13.3
Abdomen	13.3
Others	20

Once the antibiogram was obtained, the initial treatment was considered appropriate in 165 (78.9%) episodes of severe sepsis with microbiological diagnosis, and inappropriate in 39 (18.7%) episodes. The attending physician did not indicate whether the initial treatment was appropriate in the remaining five (2.4%) episodes.

### Outcome of patients

**Figure 2:**
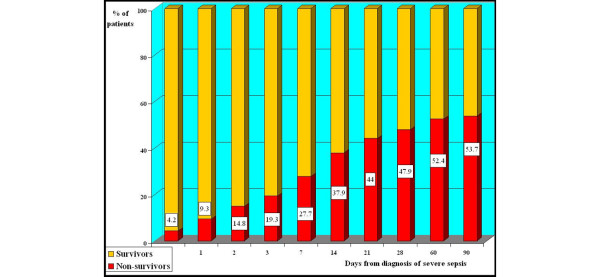
**Cumulative hospital mortality**. Numbers in squares: cumulative mortality in different days.

**Figure 3:**
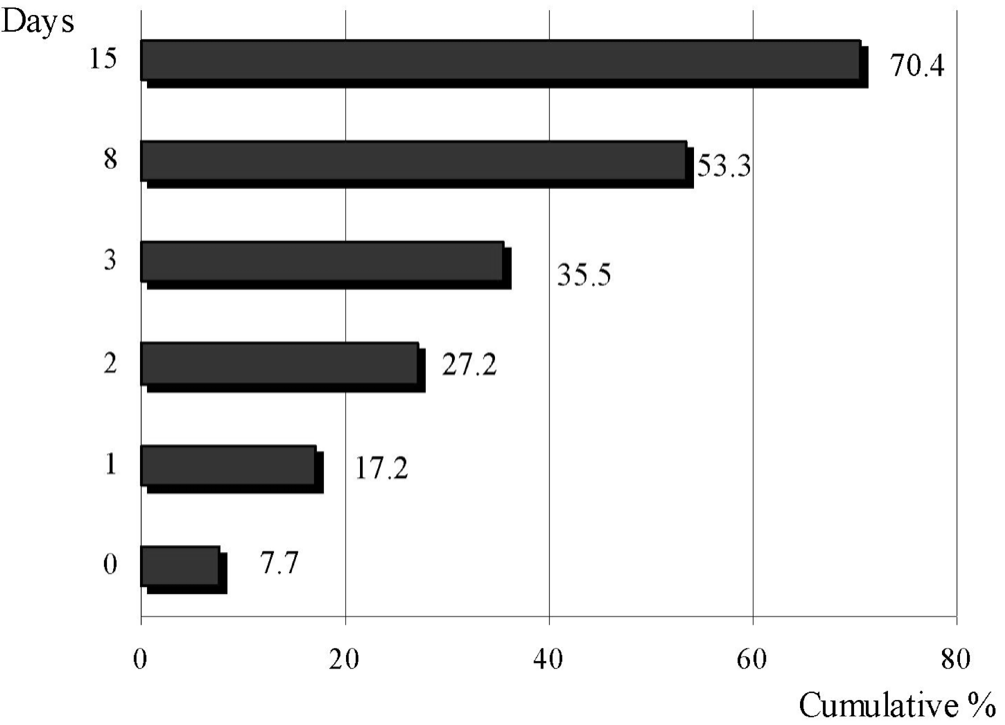
**Time course of mortality in non-survivors**. Cumulative percentage of non-survivors (n = 169) after diagnosis of severe sepsis.

The median hospital stay was 24 days (interquartile range: 11 to 44 days) and the median ICU stay was 10 days (interquartile range: 4 to 20 days). The hospital and ICU stays were significantly higher in survivors than in non-survivors (Table 3). Of the 311 patients included in the study, 169 patients (54.3%; 95% CI 48.6 to 60.0) died in the hospital and 150 (48.2%; 95% CI 42.5 to 53.9) of these died in the ICU (Table 3). Figure 2 shows the cumulative hospital mortality in the total of 311 patients. Early mortality was high, 14.8% (95% CI 10.7 to 18.9) in the first 2 days, and the mortality at 28 days was 47.9% (95% CI 42.2 to 53.6). At 90 days from diagnosis of severe sepsis, 167 patients (53.7%; 95% CI 48.0 to 59.4) had died, and 13 patients were still in hospital (4.1%), 3 in the ICU (0.9%) and 10 (3.2%) in the regular ward. Figure 3 shows the accumulated percentage of 169 non-survivors who died on the different days after inclusion in the study; 7.7% (95% CI 3.4 to 12.0) of the non-survivors died on day 0, and the accumulated mortality in the non-survivors on days 2, 8, and 15 was 27.2% (95% CI 20.2 to 34.2), 53.3% (95% CI 45.4 to 61.1) and 70.4% (95% CI 63.2 to 77.6), respectively.

**Table 3:** Outcome of 311 patients with severe sepsis

**Stay from diagnosis of severe sepsis, days, median (IQR)**				**p Value**^a^
		**Survivors**	**Non-survivors**	
		
Hospital		35 (22 to 59)	15 (7 to 30)	0.000
Intensive care unit (ICU)		12 (5 to 23)	8 (3 to 18)	0.006
Pre ICU		1.5 (0 to 7)	2 (0 to 8)	0.287
Post ICU		15 (9 to 24)	10 (4 to 28)	0.298
				
**Mortality**		**n (%)**	**95% CI**	
			
Day 28		149 (47.9)	42.2 to 53.6	
ICU		150 (48.2)	42.5 to 53.9	
Hospital		169 (54.3)	48.6 to 60.0	

	**n**	**Hospital death (%)**	**95% CI**	
	
Acquisition site:				0.655
Intra ICU	51	30 (58.8)	44.2 to 72.4	
Hospital	93	52 (55.9)	45.2 to 66.2	
Community	167	87 (52.1)	44.2 to 59.9	
Admission category:				0.715
Scheduled surgery	23	13 (56.5)	34.4 to 76.8	
Medical	179	101 (56.4)	48.9 to 64.0	
Unscheduled surgery	93	48 (51.6)	41.0 to 62.1	
Traumatological	16	7 (43.8)	19.7 to 70.1	
Infection site:				0.008
Abdomen	101	63 (62.4)	52.4 to 72.3	
Lung	138	82 (59.4)	50.9 to 68.0	
Soft tissues	8	4 (50)	15.7 to 84.3	
Surgical wound	10	3 (30)	6.7 to 65.2	
Catheter related	14	4 (28.6)	8.4 to 58.1	
Urinary tract	20	4 (20)	5.7 to 43.7	
Others	20	9 (45)	23.1 to 68.5	

^a^p Value for comparison between survivors and non-survivors.

CI, confidence interval; IQR, interquartile range.

**Figure 4:**
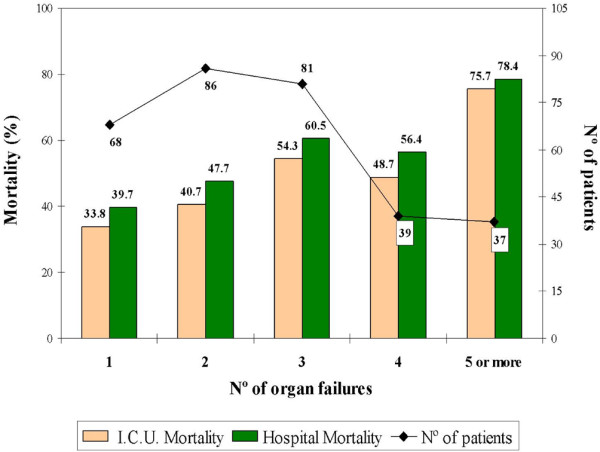
**Mortality by the number of organ failures at the time of diagnosis of severe sepsis (day 0 (D0))**. Organ failures defined according to Recombinant Human Activated Protein C Worldwide Evaluation in Severe Sepsis (PROWESS) study criteria [16]. ICU, intensive care unit.

No differences in hospital mortality rate were observed by acquisition site and admission category (Table 3). Figure 4 shows mortality by the number of organ failures at the time of severe sepsis diagnosis (D0).

### Evolution of organ dysfunction

**Figure 5:**
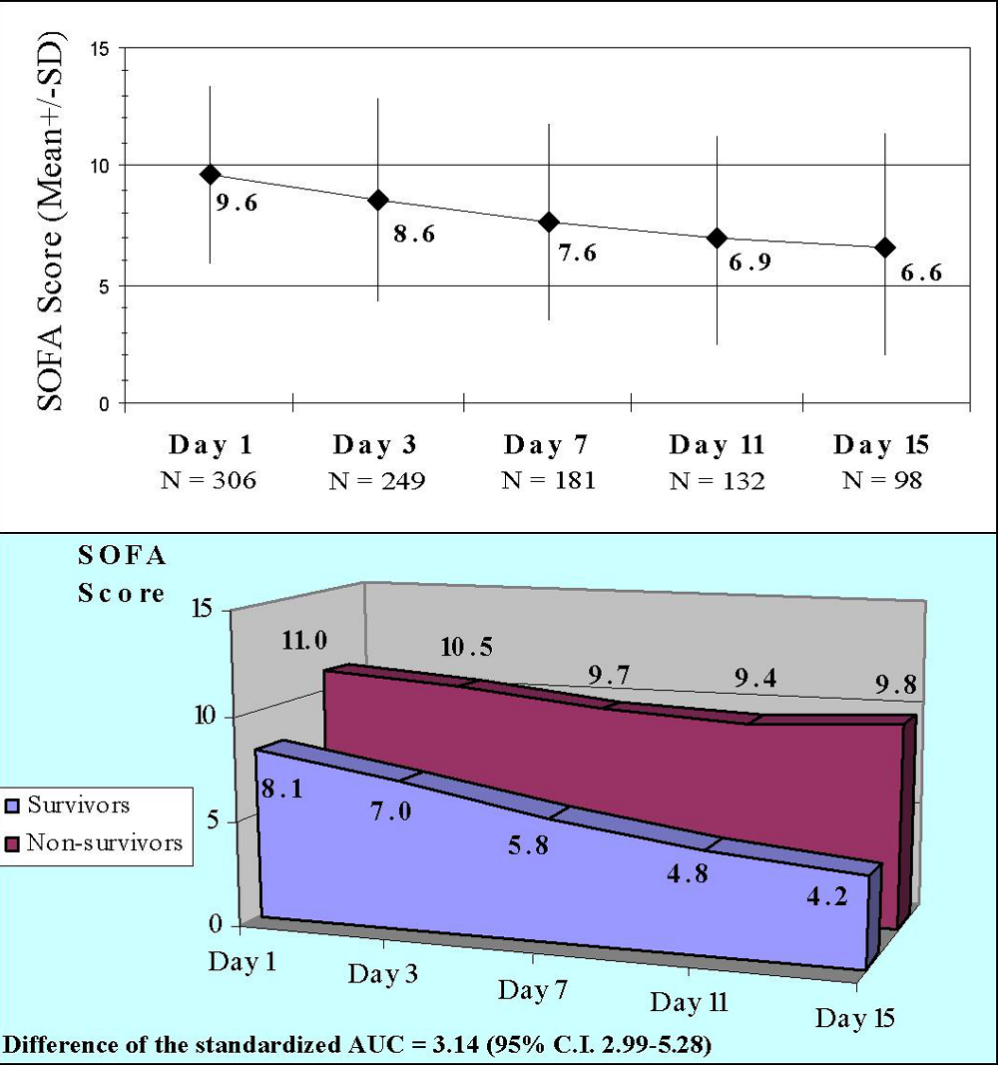
**Evolution of the SOFA score over time**. Upper panel: entire group of patients. Lower panel: area under the curve (AUC) of the Sepsis-related Organ Failure Assessment (SOFA) score trends in survivors and non-survivors. CI, confidence interval of the difference of the standardised AUC between survivors and non-survivors; SD, standard deviation.

The mean SOFA score decreased with time (9.6 points on day 1 to 6.6 points on day 15), probably because some of the patients that eventually died, who had higher scores, were still alive on day 1 (Figure 5). The mean SOFA score was initially higher in patients that died than in survivors and it remained higher throughout the first 15 days. As the time intervals between SOFA scores were not equal, to compare the evolution of SOFA scores between survivors and non-survivors over time, we calculated the standardised area under the curve for both trends and compared them. The standardised value of the area under the curve for the evolution of SOFA scores over time was 5.78 (standard error of the mean (SEM) = 0.49) in survivors and 9.92 (SEM = 0.30) in those that died. The difference between the area under the curve for those that died and those that survived was 3.14 (95% CI 2.99 to 5.28) (p < 0.001) (Figure 5).

### Risk factors for death associated with severe sepsis

The risk factors associated with death were identified in the following analyses: (a) risk factors present at diagnosis (D0) associated with early death (death within 48 h of diagnosis), (b) risk factors present at diagnosis associated with hospital mortality, and (c) risk factors associated with hospital mortality that appeared during the patient's evolution but that are not necessarily present at diagnosis.

#### Risk factors present at diagnosis (D0) associated with early death in the ICU (≤ 48 h)

In the univariate analysis, the variables that were associated with early mortality were haematological (p = 0.004) and liver failure (p = 0.005) according to the Recombinant Human Activated Protein C Worldwide Evaluation in Severe Sepsis (PROWESS) definition [16]; inappropriate initial antibiotic treatment (p = 0.03); acquisition site of the infection (p = 0.007), with early mortality higher in patients with community-acquired infections than in those that acquired the infection after admission to the ICU (25.6% vs 0%, p < 0.001), and LODS score (p = 0.02). In the multivariate analysis, the factors independently associated with early death were haematological failure, OR 1.5, (95% CI 1.3 to 3.4); liver failure, OR 2, (95% CI 1.6 to 6.3); acquisition of the infection before ICU admission, OR 2.2, (95% CI 1.0 to 4.4); and LODS score, OR 1.2, (95% CI 1.1 to 1.4).

#### Risk factors present at diagnosis (D0) associated with hospital mortality

In the univariate analysis, sex (p = 0.05), age (p = 0.003), chronic alcohol abuse (p = 0.04), congestive heart failure (p = 0.03), shock (p = 0.002), haematological (p = 0.01), neurological (p = 0.07) and liver failure (p = 0.04) according to the PROWESS definition [16], McCabe index (p < 0.0001), LODS SCORE (p < 0.0001), and the number of comorbidities (p < 0.001) were significantly associated with the risk of dying. Infection located in the urinary tract was associated with lower mortality in this subgroup of patients (p < 0.001). The multivariate analysis confirmed that both the severity of the acute organ dysfunction measured by total LODS score on day 0 and the severity of underlying conditions measured by the McCabe score were independently associated with the risk of dying, as were age and chronic alcohol abuse. Infection located in the urinary tract was independently associated with lower mortality compared to other infection sites (Table 4, Model 1).

**Table 4:** Risk factors for hospital death, present at severe sepsis diagnosis. Model 1 shows the risk factors that were independently associated with hospital death. Model 2 shows the results of a second multivariate analysis introducing the each of the variables that make up the LODS score separately

	Odds ratio	95% CI
Model 1:		
Age (< 45; 45 to 80; > 80)	3.14	1.51 to 6.54
McCabe score (for each point)	1.72	1.25 to 2.37
Chronic alcohol abuse	2.92	1.01 to 8.93
Total LODS day 0 (for each point)	1.37	1.24 to 1.51
Urinary tract origin of sepsis (pulmonary origin = 1)	0.09	0.02 to 0.52
Model 2:		
Age (< 45; 45 to 80; > 80)	4.04	1.82 to 8.93
McCabe score (for each point)	1.68	1.21 to 2.34
Neurological LODS (for each point)	1.88	1.41 to 2.52
Respiratory LODS (for each point)	1.5	1.17 to 1.93
Renal LODS renal (for each point)	1.37	1.16 to 1.61
Haematological LODS (for each point)	2.36	1.26 to 5.45
Urinary tract origin of sepsis (pulmonary origin = 1)	0.052	0.007 to 0.37

CI, confidence interval; LODS, Logistic Organ Dysfunction System.

To better analyze the impact on mortality of each organ or system dysfunction assessed by LODS, we introduced the score for each organ into the model independently; we observed that increased scores for the haematological, neurological, pulmonary, and renal components were significantly associated with mortality. Age and an increased McCabe score in comparison to the absence of prior chronic disease remained as independent risk factors for death (Table 4, Model 2).

#### Risk factors associated with hospital mortality obtained at the time of diagnosis (D0) and overtime

When overall hospital mortality was taken as the dependent variable, the variables obtained at D0 and overtime that were most strongly associated with mortality were cardiovascular dysfunction in the SOFA score on day 1 and the variable ΔSOFA 3-1, defined as the difference in the total SOFA scores on day 3 and on day 1. An increase of 1 point on the SOFA score between day 1 and day 3 increased the risk of death by a factor of 1.324. Chronic alcohol abuse, hospital stay prior to ICU admission, and APACHE II seem to be associated with the risk of death; however, the 95% confidence intervals for these variables include 1, so their association with mortality is uncertain (Table 5).

**Table 5:** Factors associated with hospital mortality present at the time of diagnosis of severe sepsis and over time

	Odds ratio	95% CI
Age	1.04	1.01 to 1.07
Site of infection:		
Lung	1	
Abdomen	1.55	0.66 to 3.70
Catheter	0.72	0.15 to 3.43
Urinary tract	0.10	0.01 to 0.78
Others	0.70	0.25 to 1.94
Chronic alcohol abuse	3.20	0.90 to 11.42
Acquisition in hospital	2.03	0.98 to 4.36
SOFA score day 1 (failure):		
Haematological	3.20	0.92 to 9.92
Cardiovascular	2.78	1.37 to 5.64
Neurological	2.39	0.76 to 7.48
Renal	1.97	0.73 to 5.27
Liver	1.67	0.46 to 6.08
Lung	1.65	0.81 to 3.36
ΔSOFA (3-1)	1.32	1.13 to 1.34
APACHE II Score	1.02	0.97 to 1.08
Pre ICU stay (days)	0.99	0.97 to 1.02

APACHE II, Acute Physiology and Chronic Health Evaluation II; CI, confidence interval; ICU: Intensive Care Unit; SOFA: Sepsis-related Organ Failure Assessment; SOFA failure: SOFA points 3 or 4.

## Discussion

The most important findings of this study were: (a) the high incidence (12.4%) of severe sepsis in the ICU and high mortality in both the ICU (48.2%) and the hospital (54.3%); (b) the association of severe sepsis with long ICU and hospital stays (median 10 days and 24 days, respectively); (c) the factors associated with early death were haematological failure and liver failure at diagnosis, acquisition of the infection prior to ICU admission and total LODS score; and (d) the factors associated with death in the hospital were age, chronic alcohol abuse, increased McCabe score, increased LODS score, ΔSOFA 3-1 and the evolution of the SOFA score.

Standardised diagnostic criteria for sepsis, severe sepsis, septic shock, and organ dysfunction and failure associated with infection [2] have enabled the epidemiological evaluation of septic syndromes, as well as of their progression in recent years and of the efficacy of new treatment measures. Using these diagnostic criteria, we found an incidence of severe sepsis of 12.4%, which is comparable to that of other series published in recent years and in line with the progression predicted by Martin *et al. *[1]. The EPISEPSIS Group [13] found an incidence of severe sepsis or septic shock of 14.6% among patients admitted to the ICU, and Finfer *et al. *found 11.8 cases of severe sepsis for every 100 admissions to the ICU [14]. Different authors have suggested that this progression might be related to the use of immunosuppressors, hospital malnutrition, alcoholism, cancer, diabetes mellitus, the growing invasiveness of both diagnostic and therapeutic measures, increased resistance of microorganisms, and the progressive aging of the population [1,9,17]. The advanced age of our population (median 68 years), the high incidence of previous immunodeficiency (13.2%), and the presence of other risk factors such as previous alcoholism, diabetes, chronic heart failure, kidney failure, liver failure, or respiratory failure confirm this increase in risk factors.

The overall incidence of severe sepsis for the population covered by the ICUs in this study was 25 cases per 100,000 inhabitants over 18 years of age per year, a figure that is lower than the incidence reported in recently published studies. This difference might be explained by seasonal bias, differences in the populations studied, differences in access to hospitals, and/or a low rate of detection of severe sepsis. The EPISEPSIS Group [14] estimated the incidence for all France at 95 cases of severe sepsis attended in the ICU per 100,000 inhabitants per year. The recruitment period in the EPISEPSIS study was only 15 days, so this high incidence might reflect a seasonal bias; however, the wide selection of hospitals and geographical areas participating in this study lend significant weight to these results. Esteban *et al. *recorded cases of sepsis admitted to 3 hospitals and estimated the overall incidence for the population at 44 cases of sepsis and 33 cases of severe sepsis attended in the ICU per 100,000 inhabitants per year [10]. The study period covered 4 randomly chosen unspecified months, which might have introduced a seasonal bias. Furthermore, the population of the geographical area assigned to the hospitals that participated in the study was mainly urban, with a high percentage of transient persons and immigrants not counted in the census. In our study, the incidence reported refers to episodes of severe sepsis among patients admitted to the ICU; the study period is wider and divided into two periods to reduce the possibility of seasonal bias on the incidence. Moreover, the population is predominantly rural (54%), with a low rate of transient persons and immigrants not counted in the census, but also with more difficulties accessing the hospitals and probably a lower rate of detection of severe sepsis before admission to the ICU.

The hospital mortality in our series (54.3%) differs from that published in the most recent series, which ranges from 28% to 48.4% [9,11,13,14,16]. However, methodological differences with our study account for much of these differences. The study by Angus *et al. *[9] is retrospective, from hospital records and also includes children. Padkin *et al. *[11] reported 47.3% mortality, but their study only includes episodes of severe sepsis that occurred during the first 24 h after ICU admission. The EPISEPSIS Study [14] reported a 60-day mortality of 41.9%, but the outcome of 11.4% of the patients who were still hospitalised 2 months after the diagnosis of severe sepsis is unknown. The control group in the PROWESS study [16] had a mortality of 31.3%; however, this study was a randomised controlled trial, so not all patients diagnosed with severe sepsis were included in the mortality analysis. Moreover, our population was older than those of the other studies (median age, 68 years vs 61 to 65 years). Ferrer *et al*. [25], in a recent article dealing with the effects on outcome of a nationwide educational intervention based on the Surviving Sepsis Campaign guidelines, reported a basal hospital mortality rate of 44%. Although slightly lower than ours, their patients had a lower age and APACHE II score. By contrast, the age of our patients was similar to those in the recent epidemiological study by Engel *et al.*, found ICU (48.4%) and hospital (55.2%) mortality similar to our series [26].

At the time of diagnosis, our patients were severely ill; this is reflected by the prevalence of multiple organ failure (78.1% had two or more organ failures and 50.4% had three or more) and the high LODS score (mean 6.3, median 6). This severity was confirmed in the first 24 h of evolution, with a high APACHE II score (median 25) and high organ dysfunction measured with the SOFA score (mean 9.6, median 10). This severity led to high early mortality: a quarter of the patients that died did so in the first 48 h and more than half of those who died did so in the first week (Figure 3). The 28-day mortality (47.9%), although high, is closer to the results obtained in the previously cited studies. Although not statistically significant, the hospital stay prior to ICU admission seems to be associated with mortality; this association, together with the initial severity of our patients, could lead to a delay in diagnosis and initial resuscitation in septic patients in other areas of the hospital. Unfortunately, our study design did not include registering the time lag between the appearance of the first signs or symptoms of sepsis and the initiation of resuscitation measures and appropriate antibiotic therapy. The importance of establishing the diagnosis and treatment early is underlined by the factors that were associated with mortality in the first 48 h in the univariate study, including community-acquired infection and inappropriate initial antibiotic treatment. Other factors, such as the origin of the patients (hospital ward, emergency department) or their classification (medical or surgical) were in agreement with other studies [14,26] and do not seem to be related to mortality. Chronic alcohol abuse was the only comorbid condition that was independently associated with increased mortality.

As in other recently published series [14,16,27], infection was most frequently located in the lungs, followed by the abdomen. Despite the increased number of Gram-positive infections registered in recent years [12,14,27], the infections found in our patients were mostly caused by Gram-negative bacilli. The isolates and the percentage of the different microorganisms are similar to those published in the European study Sepsis Occurrence in Acutely Ill Patients (SOAP) [27], and the percentage of Gram-positive cocci is identical to that recently reported by Esteban *et al. *in Spain [10]. None of the microorganisms or sites of infection was independently associated with increased mortality.

The relation between increased mortality and both the number of organ failures at diagnosis and the progression toward multiple organ failure in septic patients is well established [3-6,28]. To document both the magnitude of organ failure at diagnosis and the sequential progression of organ failures, we used two specific indices, the LODS [21] and the SOFA [22], respectively. Both scores were independently associated with mortality. Introducing the LODS into the model displaced other risk factors such as the number of organ failures, the presence of shock or respiratory failure, and the number of comorbidities (Table 4, Model 1). The mortality associated with the failure of the different organs and systems at diagnosis was more clearly delineated when each of the components of the LODS score were analyzed separately (Table 4, Model 2). Moreno *et al. *reported in 1999 that the initial SOFA score could be used to quantify the degree of organ dysfunction or failure at admission and that there was a good correlation between organ failures assessed by the maximum SOFA score and mortality [29]. Ferreira *et al. *found a correlation between increased SOFA score in the first 48 h (ΔSOFA 48-0) and mortality (OR 1.52) [30]. In our study, although the total SOFA score on day 1 was not independently associated with mortality and only one of its components (cardiovascular failure) was associated with a significant increase in the risk of death, the difference between the SOFA score on days 1 and 3 (ΔSOFA 3-1) was associated with a 1.3 times greater risk of death for each point increase in this difference. The SOFA score decreased over time in patients that survived, whereas it remained high in those that died. Comparing the area under the curve for the SOFA score obtained on days 1, 3, 5, 7, 11, and 15 showed significant differences between survivors (5.78) and non-survivors (9.92), indicating that persistent organ failure is significantly associated with mortality (Figure 5).

Our study has some limitations. First, the absence of external monitoring by independent personnel not participating in data recollection might reduce the internal validity of the study. It was not possible to carry out a random review of clinical records at each of the participating centres, although all data were reviewed in the coordinating centre and extreme values were thoroughly checked to reduce errors before introducing them into the database. Second, although this is the largest specifically designed epidemiological study of severe sepsis in the ICU in Spain and despite that the specific geographical area we have studied is large, it is difficult to extrapolate our results to the general population and obtain firm conclusions about the prognosis of patients diagnosed with severe sepsis and attended in the ICU. Nevertheless, our results can serve as a reference for future studies about this condition in our environment. Third, the effect of seasonal variation on the incidence of sepsis is well known [31]. We divided the 6-month study period in two parts comprising spring-summer and autumn-winter to minimise seasonal variation, but its effects are difficult to measure. Fourth, we began our study shortly after the results of the PROWESS [16] study were published and activated protein C had not been approved for clinical use in Spain. The use of activated protein C [16] and steroids [32] was introduced in clinical practice for the treatment of severe sepsis after all the data for our study had been compiled. Subsequently, the Surviving Sepsis Campaign was launched, the SSC Clinical Practice Guidelines [18] were published, and the implementation of the resuscitation and management bundles were promoted by the SSC and the Institute for Healthcare Improvement (IHI). It is likely that the use of activated protein C and the application of the measures promoted by the SSC and IHI have reduced the morbidity and mortality of septic patients admitted to the ICU after our study. Thus, our results can also be useful as a reference for future studies about the impact of these measures on the outcome of patients with severe sepsis admitted to the ICU in our setting. Fifth, although the data registration forms included a section for reporting the decision to limit treatment and withdraw life support, the lack of a valid unified 'do not resuscitate' protocol for all the ICUs participating in the study makes it impossible to reach conclusions about the influence of this type of decisions on hospital mortality in our series.

## Conclusion

We found an incidence of severe sepsis attended in the ICU of 12.4%, an estimated incidence of 25 cases of severe sepsis for every 100,000 inhabitants per year for the overall population, high ICU and hospital mortality (48.2% and 54.3%, respectively), and a prolonged hospital stay (median 24 days). Two or more organ failures were present at diagnosis in 78.1% of patients and 27.2% of those that died did so in the first 48 h, which suggests delays in diagnosis, in initial resuscitation, and in beginning appropriate antibiotic treatment. Gram-negative bacteria caused 50% of the infections and pneumonia was the most common cause of severe sepsis in our environment. The LODS score was a good prognostic factor when determined at the moment of diagnosis. The evolution of organ failure assessed by the SOFA score was significantly different in survivors and non-survivors. The implementation of new treatment strategies in recent years and the development of the SSC measures for early diagnosis and resuscitation aimed at significantly reducing mortality in severe sepsis and septic shock necessitate new epidemiological studies to enable us to evaluate the efficacy of these measures.

## Key messages

• In this prospective, observational, multicentre cohort study, we found a high incidence of severe sepsis in the ICU and high ICU and hospital mortality, and an association of severe sepsis with long ICU and hospital stays.

• The high prevalence of multiple organ failure at diagnosis and the high mortality in the first 48 h suggests delays in diagnosis, in initial resuscitation, and/or in beginning appropriate antibiotic treatment.

• The LODS score and the SOFA score were independently associated with mortality. The LODS score was a good prognostic factor when determined in the day of diagnosis of severe sepsis.

## Abbreviations

APACHE II: Acute Physiology and Chronic Health Evaluation II; AUC: area under the curve; BAL: bronchoalveolar lavage; CHF: congestive heart failure; CI: confidence interval; GCS: Glasgow Coma Score; GNB: Gram negative bacilli; GPC: Gram positive cocci; ICU: Intensive Care Unit; IDDM: insulin-dependent diabetes mellitus; IHI: Institute for Healthcare Improvement; INE: Instituto Nacional de Estadística (National Statistics Institute); IQR: interquartile range; LODS: Logistic Organ Dysfunction System; OR: odds ratio; SD: standard deviation; SEM: standard error of the mean; SIRS: systemic inflammatory response syndrome; SOFA: Sepsis-related Organ Failure Assessment; SSC: Surviving Sepsis Campaign.

## Competing interests

The authors declare that they have no competing interests.

## Authors' contributions

The study was designed by JB and AM-B. They also initiated and coordinated the study, participated in the screening of patients, acquisition, analysis and interpretation of data, and they drafted and revised critically the manuscript. JV was involved in analysis and interpretation of data and he revised the manuscript critically for important intellectual content. VS, FT, FG, LT, JC, AG-L, DC, MV, MDeF, MJL, AC, JG and BA participated in study design, screening of patients and acquisition of data. AM was involved in analysis and interpretation of data and he made the principal statistical analysis. All members of the Grupo de Estudios y Análisis en Cuidados Intensivos (GRECIA) participated in the screening of patients and acquisition of data. All authors read and approved the final manuscript.

## Supplementary Material

Additional file 1 A Word file listing the Members of Grupo de Estudios y Análisis en Cuidados Intensivos (GRECIA). Click here for file

Additional file 2 A Word file showing the definitions for intensive care unit (ICU) type, patient categories, comorbidities, systemic inflammatory response syndrome, sepsis, severe sepsis, septic shock and organ dysfunction. Click here for file

## References

[B1] Martin GS, Mannino DM, Eaton S, Moss M (2003). The epidemiology of sepsis in the United States from 1979 through 2000. N Engl J Med.

[B2] Bone RC, Balk RA, Cerra FB, Dellinger RP, Fein AM, Knaus WA, Schein RM, Sibbald WJ (1992). Definition for sepsis and organ failure and guidelines for use of innovative therapies in sepsis. The ACCP/SCCM Consensus Conference Committee. American College of Chest Physicians/Society of Critical Care Medicine. Chest.

[B3] Brun-Buisson C (2000). The epidemiology of the systemic inflammatory response. Intensive Care Med.

[B4] Barriere SL, Lowry SF (1995). An overview of mortality risk prediction in sepsis. Crit Care Med.

[B5] Brun-Buisson C, Doyon F, Carlet J, Dellamonica P, Gouin F, Lepoutre A, Mercier JC, Offenstadt G, Régnier B (1995). Incidence, risk factors, and outcome of severe sepsis and septic shock in adults. A multicenter prospective study in intensive care units. French ICU Group for Severe Sepsis. JAMA.

[B6] Rangel-Frausto MS, Pittet D, Costigan M, Hwang T, Davis CS, Wenzel RP (1995). The natural history of the systemic inflammatory response syndrome (SIRS): a prospective study. JAMA.

[B7] Sands KE, Bates DW, Lanken PN, Graman PS, Hibberd PL, Kahn KL, Parsonnet J, Panzer R, Orav EJ, Snydman DR, Black E, Schwartz JS, Moore R, Johnson BL, Platt R, Academic Medical Center Consortium Sepsis Project Working Group (1997). Epidemiology of sepsis syndrome in 8 academic medical centers. JAMA.

[B8] Salvo I, de Cian W, Musicco M, Langer M, Piadena R, Wolfler A, Montani C, Magni E (1995). The Italian sepsis study: preliminary results on the incidence and evolution of SIRS, sepsis, severe sepsis and septic shock. Intensive Care Med.

[B9] Angus DC, Linde-Zwirble WT, Lidicker J, Clermont G, Carcillo J, Pinsky MR (2001). Epidemiology of severe sepsis in the United States: analysis of incidence, outcome, and associated costs of care. Crit Care Med.

[B10] Esteban A, Frutos-Vivar F, Ferguson ND, Peñuelas O, Lorente JA, Gordo F, Honrubia T, Algora A, Bustos A, García G, Díaz-Regañón IR, de Luna RR (2007). Sepsis incidence and outcome: contrasting the intensive care unit with the hospital ward. Crit Care Med.

[B11] Padkin A, Goldfrad C, Brady AR, Young D, Black N, Rowan K (2003). Epidemiology of severe sepsis occurring in the first 24 hrs in intensive care units in England, Wales, and Northern Ireland. Crit Care Med.

[B12] Annane D, Aegerter P, Jars-Guincestre MC, Guidet B, CUB-Réa Network (2003). Current epidemiology of septic shock: the CUB-Réa Network. Am J Respir Crit Care Med.

[B13] Finfer S, Bellomo R, Lipman J, French C, Dobb G, Myburgh J (2004). Adult-population incidence of severe sepsis in Australian and New Zealand intensive care units. Intensive Care Med.

[B14] Brun-Buisson C, Meshaka P, Pinton P, Vallet B, EPISEPSIS Study Group (2004). EPISEPSIS: a reappraisal of the epidemiology and outcome of severe sepsis in French intensive care units. Intensive Care Med.

[B15] Alberti C, Brun-Buisson C, Burchardi H, Martin C, Goodman S, Artigas A, Sicignano A, Palazzo M, Moreno R, Boulmé R, Lepage E, Le Gall R (2002). Epidemiology of sepsis and infection in ICU patients from an international multicentre cohort study. Intensive Care Med.

[B16] Bernard GR, Vincent JL, Laterre PF, LaRosa SP, Dhainaut JF, Lopez-Rodriguez A, Steingrub JS, Garber GE, Helterbrand JD, Ely EW, Fisher CJ, Recombinant human protein C Worldwide Evaluation in Severe Sepsis (PROWESS) study group (2001). Efficacy and safety of recombinant human activated protein C for severe sepsis. N Engl J Med.

[B17] Angus DC, Wax RS (2001). Epidemiology of sepsis: an update. Crit Care Med.

[B18] Dellinger RP, Carlet JM, Masur H, Gerlach H, Calandra T, Cohen J, Gea-Banacloche J, Keh D, Marshall JC, Parker MM, Ramsay G, Zimmerman JL, Vincent JL, Levy MM (2004). Surviving Sepsis Campaign Guidelines for management of severe sepsis and septic shock. Intensive Care Med.

[B19] McCabe WR, Jackson GG (1962). Gram negative bateremia: etiology and ecology. Arch Intern Med.

[B20] Knaus WA, Draper EA, Wagner DP, Zimmerman JE (1985). APACHE II: a severity of disease classification system. Crit Care Med.

[B21] Le Gall JR, Klar J, Lemeshow S, Saulnier F, Alberti C, Artigas A, Teres D (1996). The Logistic Organ Dysfunction system. A new way to assess organ dysfunction in the intensive care unit. ICU Scoring Group. JAMA.

[B22] Vincent JL, Moreno R, Takala J, Willatts S, De Mendonça A, Bruining H, Reinhart CK, Suter PM, Thijs LG (1996). The SOFA (Sepsis Related Organ Failure Assessment) score to describe organ/dysfunction/failure. On behalf of the Working Group on Sepsis-Related Problems of the European Society of Intensive Care Medicine. Intensive Care Med.

[B23] Garner JS, Jarvis WR, Emori TG, Horan TC, Hughes JM (1988). CDC definitions for nosocomial infections. Am J Infect Control.

[B24] Instituto Nacional de Estadística (INE) Censo de Población y Viviendas 2001. http://www.ine.es/jaxi/menu.do?type=pcaxis&path=%2Ft20%2Fe242&file=inebase&L=.

[B25] Ferrer R, Artigas A, Levy MM, Blanco J, González-Díaz G, Garnacho-Montero J, Ibáñez J, Palencia E, Quintana M, de la Torre-Prados MV, Edusepsis Study Group (2008). Improvement in process of care and outcome after a multicenter severe sepsis educational program in Spain. JAMA.

[B26] Engel C, Brunkhorst FM, Bone HG, Brunkhorst R, Gerlach H, Grond S, Gruendling M, Huhle G, Jaschinski U, John S, Mayer K, Oppert M, Olthoff D, Quintel M, Ragaller M, Rossaint R, Stuber F, Weiler N, Welte T, Bogatsch H, Hartog C, Loeffler M, Reinhart K (2007). Epidemiology of sepsis in Germany: results from a national prospective multicenter study. Intensive Care Med.

[B27] Vincent JL, Sakr Y, Sprung CL, Ranieri VM, Reinhart K, Gerlach H, Moreno R, Carlet J, Le Gall JR, Payen D, Sepsis Occurrence in Acutely Ill Patients Investigators (2006). Sepsis in European intensive care units: results of the SOAP Study. Crit Care Med.

[B28] Alberti C, Brun-Buisson C, Chevret S, Antonelli M, Goodman SV, Martin C, Moreno R, Ochagavia AR, Palazzo M, Werdan K, Le Gall JR, European Sepsis Study Group (2005). Systemic inflammatory response and progresion to severe sepsis in critically ill infected patients. Am J Respir Crit Care Med.

[B29] Moreno R, Vincent JL, Matos R, Mendonça A, Cantraine F, Thijs L, Takala J, Sprung C, Antonelli M, Bruining H, Willatts S (1999). The use of maximum SOFA score to quantify organ dysfunction/failure in intensive care. Results of a prospective, multicentre study. Intensive Care Med.

[B30] Ferreira FL, Bota DP, Bross A, Mélot C, Vincent JL (2001). Serial evaluation of the SOFA score to predict outcome in critically ill patients. JAMA.

[B31] Danai PA, Sinha S, Moss M, Haber MJ, Martin GS (2007). Seasonal variation in the epidemiology of sepsis. Crit Care Med.

[B32] Annane D, Sébille V, Charpentier C, Bollaert PE, François B, Korach JM, Capellier G, Cohen Y, Azoulay E, Troché G, Chaumet-Riffaut P, Bellissant E (2002). Effect of treatment with low doses of hydrocortidsone and fludrocortisone on mortality in patients with septic shock. JAMA.

