# Supplementary material for: Incidence, organ dysfunction and mortality in severe sepsis: a Spanish multicentre study
Source: Crit Care. 2008 Dec 17;12(6):R158. doi: 10.1186/cc7157 (PMC2646323; doi:10.1186/cc7157)
Supplement: Additional file 1 — A Word file listing the Members of Grupo de Estudios y Análisis en Cuidados Intensivos (GRECIA). [file cc7157-S1.doc]

**Appendix 1**

***Members of Grupo de Estudios y Análisis en Cuidados Intensivos (G.R.E.C.I.A.):***

Jesús Blanco, Arturo Muriel-Bombín, Luis Ángel Domínguez *(Hospital Universitario Río Hortega. Valladolid, Spain)*. Víctor Sagredo, Juan Carlos Ballesteros *(Hospital Clínico Universitario de Salamanca. Salamanca, Spain)*. Francisco Taboada, Guillermo Muñiz *(Hospital Central de Asturias. Oviedo, Spain)*. Francisco Gandía, Felipe Bobillo *(Hospital Clínico Universitario de Valladolid. Valladolid, Spain)*. Luis Tamayo, Javier Collado *(Hospital Río Carrión. Palencia, Spain)*. Angel García-Labattut *(Hospital General de Soria. Soria, Spain)*. Demetrio Carriedo *(Complejo Hospitalario de León. Leon, Spain)*. Manuel Valledor, María Teresa Antuña *(Hospital de San Agustín. Avilés, Spain)*. Martín De Frutos *(Hospital General Yagüe. Burgos, Spain)*. María Jesús López, José Joaquín Cortina *(Hospital General de Segovia. Segovia, Spain)*. Teresa Saldaña, Ana Caballero, Teresa Alvarez *(Hospital Virgen de la Concha.. Zamora, Spain)*. José Guerra *(Hospital de Cabueñes. Gijón, Spain)*. Braulio Álvarez, J. José Sandoval *(Hospital del Bierzo. Ponferrada, Spain)*.
