# Supplementary material for: Incidence, organ dysfunction and mortality in severe sepsis: a Spanish multicentre study
Source: Crit Care. 2008 Dec 17;12(6):R158. doi: 10.1186/cc7157 (PMC2646323; doi:10.1186/cc7157)
Supplement: Additional file 2 — A Word file showing the definitions for intensive care unit (ICU) type, patient categories, comorbidities, systemic inflammatory response syndrome, sepsis, severe sepsis, septic shock and organ dysfunction. [file cc7157-S2.doc]

**Appendix 2**

**Definitions**

***A. ICU type*.** Participating ICUs were classified as: a) Medical, if 80% or more patients were medical; b) Surgical, if 80% or more patients were surgical; c) Mixed medical-surgical, if both medical and surgical patients were treated (<80% and >20% from each group); d) Cardiac surgical, if 80% or more patients came from heart surgery; d) Traumatological, if 80% or more patients were traumatological; e) Neurological-neurosurgical, if 80% or more patients were neurological or neurosurgical.

***B. Patient categories.*** Patients were classified as: a) Medical, i.e., not operated on or operated on more than 10 days before or after ICU admission; b) Surgical, i.e. operated on in the 10 days immediately before or after ICU admission; c) Traumatological, i.e., admitted to the ICU for trauma, regardless of whether they were operated on. Surgical patients were further defined as: a) urgent, when they were not listed on the surgery schedule the day before the operation, or b) planned, when they were listed on the surgery schedule the day before the operation. All patients admitted to the ICU for reanimation who underwent urgent surgery after reanimation were considered surgical patients, regardless of their origin.

***C. Comorbidities.*** The following clinical conditions prior to inclusion in the study were recorded: **Immunodeficiency**: patients a) that received immunosuppressors, chemotherapy, or radiotherapy; b) diagnosed with leukemia, lymphoma, or AIDS; c) that received high doses of glucocorticoids in the 3 months prior to ICU admission. **AIDS**: HIV-positive patients a) hospitalized for pneumonia or *Pneumocystis jirovecii*, Kaposi’s sarcoma, lymphoma, toxoplasmosis, or tuberculosis; b) with CD4 <50/mm^3^. **Transplanted patients**: kidney, liver, bone marrow, pancreas, lung, or heart. **Metastatic cancer**: metastases proven at imaging, surgery, or by tumor markers. **Chronic heart failure:** Class IV on the New York Heart Association (NYHA) classification. **Chronic renal failure:** patients included in hemodialysis or chronic peritoneal dialysis programs. **Chronic respiratory failure**: patients a) with obstructive, restrictive, or vascular disease that restricts daily activity; b) with documented chronic hypoxia; c) receiving home or ambulatory oxygen therapy, or d) with secondary polycythemia or severe pulmonary hypertension (>40 mmHg). **Chronic liver failure:**  biopsy-proven cirrhosis of the liver with portal hypertension documented by a history of gastrointestinal bleeding or liver failure, hepatic encephalopathy, or coma. **Insulin-dependent diabetes mellitus:** hyperglycemia as a consequence of defects in insulin secretion, in the action of insulin, or both that requires periodic administration of exogenous insulin. **Chronic alcohol abuse:** in men, defined as the ingestion of >14 drinks per week (equivalent to 400 ml wine or 750 ml beer daily); in women, defined as the ingestion of >7 drinks per week (equivalent to 200 ml wine or 375 ml beer daily). **Hypercoagulabilty syndrome:** a) hereditary deficit of Protein C, Protein S, or Antithrombin III, b) the presence of anticardiolipin or antiphospholipid antibodies, lupus anticoagulant or homocysteinemia, or c) deep vein thrombosis or pulmonary thromboembolism in the three months prior to the episode of severe sepsis. **Increased risk of bleeding**: a) evidence of active bleeding in the postoperative period; b) history of hemorrhagic diathesis or gastrointestinal bleeding in the 6 weeks before the episode, if not corrected surgically; c) trauma leading to increased risk of bleeding; d) head trauma, intracranial mass of any type, prior intracranial surgery, e) any prior ischemic or hemorrhagic process in the central nervous system; f) any surgical intervention in the 12 hours before the episode of severe sepsis.

***D. Infection:*** History, symptoms, examination, and laboratory findings suggestive of an infection that would justify antibiotic therapy. Depending on the evidence, infection was classified as: **Suspected:** sufficient evidence to begin antibiotic therapy. **Clinically documented:** clinical presentation compatible with infection and pus seen at aspiration, puncture, or surgery or signs at diagnostic imaging without microbiological documentation. **Documented microbiologically:** infection in which germs were cultivated and identified in at least one tissue or body-fluid culture and treated with antibiotics. **Community-acquired:** an infection that was present on admission to the hospital or that developed within 48 after admission. **Nosocomial:** an infection that was absent on admission and developed more than 48 after admission to hospital or in the ICU.

***E. SYSTEMIC INFLAMMATORY RESPONSE SYNDROME (SIRS) and SEPSIS*** were defined according to the criteria established at the 1991 Consensus Conference [2].

***F. SEVERE SEPSIS*** was defined as at least one recent organ dysfunction associated with the onset of sepsis.

***G. ORGAN DYSFUNCTION:*** In addition to the progression of organ failure during the patient’s evolution (SOFA, see above), we recorded the organs and systems that had failed at the time of inclusion (D0) according to the following criteria, which were defined in the PROWESS study [16]: **Cardiovascular.** Shock: systolic arterial pressure ≤90 mmHg or mean arterial pressure ≤70 mmHg, during at least 1 hour despite adequate resuscitation with fluids or adequate intravascular volume; or use of vasopressors (dopamine ≥5 µg/Kg/minute; noradrenalin or adrenalin at any dose; dobutamine was not taken into account). Unexplained metabolic acidosis (pH <7.30 or base excess ≤-5 mmol/l) associated with an arterial lactate concentration ≥ 2 mmol/l with no other apparent cause. **Respiratory**: mechanical ventilation; or PaO2/FiO2 <250 mmHg if other organ dysfunction was present; or PaO2/ FIO2 < 200 mmHg if only pulmonary dysfunction was present. **Nervous system**: encephalopathy with GCS <13 without sedation unexplained by other causes. **Kidney**: diuresis <0.5 ml/Kg/hour despite adequate blood volume, or creatinine >1.9 mg/dl. **Liver**: bilirubin >3 mg/dl or prothrombin time <50% related to a hepatic cause. **Hematological**: platelets <80,000/ml^3^ or decrease of 50% in the 3 previous days.
